# Comparative Evaluation of Tissue Adhesives and Sutures in the Management of Facial Laceration Wounds in Children

**DOI:** 10.3390/jpm13091350

**Published:** 2023-08-31

**Authors:** Yu-Chi Tsai, Dun-Wei Huang, Yu-Yu Chou, Yu-Chin An, Yung-Sheng Cheng, Po-Huang Chen, Yuan-Sheng Tzeng

**Affiliations:** 1 Division of Plastic and Reconstructive Surgery, Department of Surgery, Tri-Service General Hospital, National Defense Medical Center, Taipei 11490, Taiwan; yuchi220729@gmail.com (Y.-C.T.); gdream12731@gmail.com (D.-W.H.); justyu1112@gmail.com (Y.-Y.C.); 2 Division of Traumatology, Department of Surgery, Tri-Service General Hospital, National Defense Medical Center, Taipei 11490, Taiwan; 3 Department of Emergency Medicine, Tri-Service General Hospital, National Defense Medical Center, Taipei 11490, Taiwan; silverstaryu@gmail.com; 4 Division of Pediatric Surgery, Department of Surgery, Tri-Service General Hospital, National Defense Medical Center, Taipei 11490, Taiwan; happyglenndmc.yungsheng@gmail.com; 5 Department of Internal Medicine, Tri-Service General Hospital, National Defense Medical Center, Taipei 11490, Taiwan; 6 Department of Surgery, Zuoying Branch of Kaohsiung Armed Forces General Hospital, Kaohsiung 813204, Taiwan

**Keywords:** tissue adhesives, suture, wound, laceration, child

## Abstract

Background: This study evaluated tissue adhesives in comparison to sutures for treating facial lacerations in children. Methods: We retrospectively analyzed data from September 2017 to August 2022 involving pediatric facial lacerations managed with either tissue adhesives or sutures. Results: Among 50 children, 20 received tissue adhesives, and 30 received sutures. Both methods showed comparable outcomes in terms of wound complications such as dehiscence (adjusted odds ratio = 1.56, 95% CI = 0.08–31.25) and infection (adjusted odds ratio = 2.17, 95% CI = 0.08–58.80). The cosmetic outcomes, assessed using the Hollander Wound Evaluation Score, were also consistent between groups (adjusted beta = −0.55, 95% CI = −1.15–0.05). Notably, those treated with tissue adhesives reported greater satisfaction (adjusted beta = 1.13, 95% CI = 0.63 −1.63) and experienced significantly less pain (adjusted beta = −3.03, 95% CI = −4.15–−1.90). Conclusions: Both techniques displayed similar rates of infection, dehiscence, and cosmetic outcomes. However, tissue adhesives were associated with increased patient comfort, especially in terms of reduced pain and greater satisfaction.

## 1. Introduction

Managing facial lacerations in pediatric patients presents a multifaceted challenge, encompassing pain management and emotional distress. A considerable proportion of these injuries, necessitating wound closure, find their way to emergency departments. The fundamental objective of wound management is to achieve hemostatic closure while enhancing cosmetic outcomes and curtailing complications, notably infection [1]. Choosing the optimal repair method is pivotal, with choices spanning sutures, staples, adhesive tapes, and octylcyanoacrylate (OCA) tissue adhesives. Suturing remains the prevailing technique for mending facial cuts, yet the outcome also depends on the operator’s skill [2], requires sterilized instruments, carries the risk of needlestick to the operator, and requires suture removal [3]. Alongside this, the prevalence of needle phobia among children further compounds the challenge, engendering anxiety and discomfort during medical procedures involving injections [4].

Sutures offer a robust closure mechanism, particularly fitting for larger or deeper wounds. They enable precise alignment of wound edges, ensuring optimal healing. However, this technique is time-intensive and requires skilled practitioners to yield favorable cosmetic outcomes. The potential necessity for local anesthesia introduces pain and anxiety, further amplified in pediatric cases. Sterilized instruments are imperative, and there exists a perpetual risk of needlestick injuries to healthcare professionals. Moreover, the subsequent suture removal procedure can be distressing and uncomfortable for patients [3]. These combined factors, coupled with the innate fear of needles among children, exacerbate stress for both young patients and their caregivers.

In contrast, OCA tissue adhesives offer an array of benefits for wound closure. Originating in 1949, these adhesives are topically applied to the epidermis, polymerizing upon contact with tissue anions to form a bonding film that aligns tissue edges when appropriately positioned. Unlike sutures, tissue adhesives are swift to apply and virtually painless, obviating the need for local anesthesia injections. This characteristic holds particular relevance in pediatrics, circumventing needle-associated distress. Furthermore, there is no requirement for suture removal, minimizing patient discomfort during follow-up. The waterproof nature of tissue adhesives allows patients to shower without jeopardizing wound integrity [5]. However, these adhesives carry the risk of complications, encompassing infection, dehiscence, and local reactions such as erythema, edema, and pain [6,7,8].

This article is dedicated to the intricate realm of pediatric facial laceration management, emphasizing the need for judicious repair method selection. It sheds light on the challenges posed by conventional sutures, encompassing pain, time consumption, needle-related distress, and potential complications. Concurrently, it underscores the merits of tissue adhesives, such as pain-free application, diminished distress, elimination of suture removal, and water resistance. However, these advantages come paired with their own set of complications. The study in focus aims to compare outcomes between OCA tissue adhesives and sutures in the repair of facial cutaneous lacerations in children, encompassing factors such as cosmetic appearance and patient-reported experiences.

## 2. Materials and Methods

In this study, we reviewed our experience with the closure of clean facial laceration wounds in children using either tissue adhesives or standard sutures from September 2017 to August 2022. Data were collected through a retrospective review of medical records from the emergency and outpatient departments of Tri-Service General Hospital, a tertiary care facility in Taiwan, as well as through follow-up phone calls three months after outpatient visits.

The study was approved by the Institutional Review Board of Tri-Service General Hospital in Taipei, Taiwan (TSGHIRB no. A202205127), and informed consent was waived due to the de-identified nature of the data collected through chart review.

### 2.1. Patients

The following were the inclusion criteria for participants in the study: children aged 1–18 years, in generally good health with no significant systemic abnormalities, who returned for follow-up within 7 days and 3 months, and had a laceration wound length of less than 5 cm, no more than 4–8 mm deep, requiring the use of nylon size 5-0 or smaller sutures for skin closure, as determined by the physician. The choice of suture size was based on the comparable functional tensile strength of OCA tissue adhesives with 5-0 sutures (as per Dimensional Analysis Systems, Inc., Troy, MI, USA).

The following were the exclusion criteria: participants with a history of peripheral vascular disease, multiple traumas, insulin-dependent diabetes mellitus, a bleeding diathesis, or a known allergy to cyanoacrylate compounds or formaldehyde were excluded. In addition, wounds caused by animal or human bites, punctures, decubitus ulcers, or crush injuries resulting in a burst (stellate) laceration were not included in the study. Furthermore, wounds with visible signs of infection, gangrene, contamination or devitalized tissue, or active rashes, as well as wounds located on the vermilion border of the lip or within the mucosa, were also excluded.

In this study, a structured data sheet with closed-ended questions was used to record patient and wound characteristics. The data collected included patient demographics such as age, sex, height, weight, and body mass index (BMI); medical history, including comorbidities and medications; wound characteristics such as location, length, and the status of the tissue adhesive used; and information on the wound closure technique employed.

### 2.2. Evaluation

SurgiSeal topical skin adhesive, a type of tissue adhesive, is supplied in a single-use, sterile plastic vial and contains 0.35 mL of octyl-2-cyanoacrylate tissue adhesive [9]. To use, hold the applicator with the applicator sponge facing upward, snap the tip along the perforated line, and fold over. Then, gently squeeze the liquid SurgiSeal adhesive from the applicator onto the sponge. As the wound edges are carefully brought together by the operator or assistant, apply the adhesive in two light coats, using a light brush stroke motion. Allow 30 s between the first and second applications. Once the final coat is applied, maintain a manual approximation of the wound edges for about 60 s. Avoid introducing the adhesive between the wound edges, as this may interfere with healing. For the control group, wounds were closed using standard techniques with nylon size 5-0 or smaller sutures [3,10].

The outcome measures of this study included complications, cosmetic appearance, and patient-reported outcomes. All wounds were re-evaluated within 7 days in the outpatient department for signs of infection and dehiscence. If a patient was prescribed antibiotics specifically for the wound infection, the wound was considered infected. The overall clinical assessment of infection had a high interobserver agreement and was considered reliable [11]. Wound dehiscence is defined as either a partial or total separation of wound edges that were previously approximated, generally suggesting a failure in the wound healing process. In situations where dehiscence was observed, it was characterized by the necessity to re-approximate the wound using a wound closure device [12].

The cosmetic appearance of the wounds was evaluated 3 months after closure using the Hollander Wound Evaluation Score (HWES), which is a previously validated 6-item ordinal scale [11,13]. Using the HWES, wounds were assessed based on six criteria: step-off of borders, contour irregularities, margin separation, edge inversion, excessive distortion, and overall appearance. Each item is assigned one point, with a possible total of 6 points. In this scoring system, a lower score is indicative of a better cosmetic outcome. Therefore, a score closer to zero represents the best possible cosmetic appearance, while a score of six indicates significant deviations from the ideal outcome.

The patient-reported outcomes were assessed through a questionnaire that included satisfaction and pain. The visual analog scale, a psychometric response scale ranging from zero to ten, was used to measure the extent of satisfaction and pain. The scale was designed with “not satisfied” or “no pain” corresponding to a score of 0 and “very satisfied” or “pain too intense to be tolerated” corresponding to a score of 10 [14].

### 2.3. Statistical Analysis

The descriptive information of continuous and categorical variables was presented in terms of means (standard deviations) and frequencies (percentages), respectively. To compare the characteristics and covariates, Student’s *t*-tests and chi-squared tests were used for continuous and categorical variables, respectively. The odds ratio (OR) with a 95% confidence interval (CI) was estimated by comparing the use of tissue adhesives and conventional sutures through multivariate logistic and linear regression analysis, taking into account covariates such as age, sex, BMI, history of eczema and keloid, and wound incision length. A two-sided *p*-value of <0.05 was considered statistically significant. The statistical analysis was performed using SPSS software (Version 22.0 for Windows, IBM Inc., Chicago, IL, USA).

## 3. Results

### 3.1. Patients’ Characteristics

**Figure 1:**
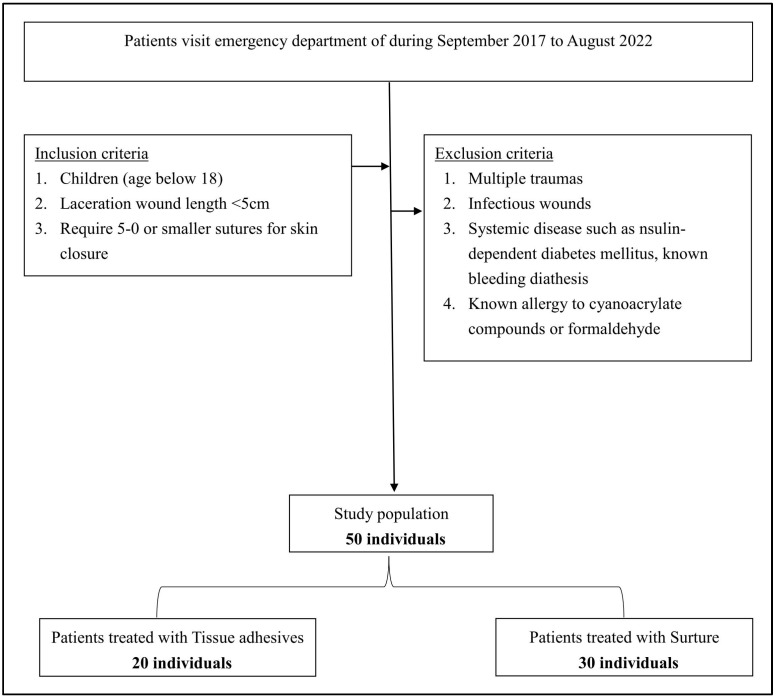
Flow diagram of patient selections.

Fifty patients were included in the study, with twenty patients receiving topical skin adhesives (tissue adhesives) and thirty patients receiving sutures (suture group) (Figure 1).

The mean age for the tissue adhesive group was 5 years with a standard deviation of 3.34, while for the suture group, it was 4.77 years with a standard deviation of 3.13. The proportion of male patients in the tissue adhesive group was 60% and 56.7% in the suture group. Other recorded patient characteristics included body height, body weight, BMI, history of eczema and keloid, and previous soft tissue infection. However, no significant difference was noted between the two groups in terms of these characteristics (Table 1).

### 3.2. Wound Condition

We recorded the characteristics of the wound location (50.0% chin, 10.0% eyebrow, 35.0% forehead, and 5.0% lower eyelid for the tissue adhesive group and 50.0% chin, 10.0% eyebrow, 33.3% forehead, and 6.7% lower eyelid for the suture group), wound incision length (1.48 ± 0.63 cm for the tissue adhesive group and 1.32 ± 0.47 cm for the suture group), and the initial status of the tissue adhesive (5.0% completely sloughed off and 95.0% intact for the tissue adhesive group and 0% completely sloughed off and 100% intact for the suture group). No significant differences were found between the two groups regarding the wound conditions (Table 1).

### 3.3. Outcome: Complications

Table 2 showed that there was no significant difference between the tissue adhesive and suture groups in terms of wound dehiscence (5.0% *vs.* 0%, *p* = 0.400) and wound infection (5.0% *vs.* 6.7%, *p* = 1.000). In order to compare the rates of complications between the two groups, unadjusted and adjusted logistic regression analyses were performed (Table 3). The results showed that there was no significant difference in the unadjusted models (wound dehiscence: OR = 1.53, 95% CI = 0.09–25.90, *p* = 0.770; wound infection: OR = 0.74, 95% CI = 0.06–8.71, *p* = 0.809) and adjusted models (wound dehiscence: adjusted OR = 1.56, 95% CI = 0.08–31.25, *p* = 0.773; wound infection: adjusted OR = 2.17, 95% CI = 0.08–58.80, *p* = 0.645).

### 3.4. Outcome: Cosmetic Appearance

Table 2 revealed no significant difference between the tissue adhesive and suture groups regarding the cosmetic appearance score as measured by the HWES (0.20 ± 0.52 *vs.* 0.77 ± 1.22, *p* = 0.054). To investigate the cosmetic appearance outcomes between the two groups, unadjusted and adjusted logistic regression analyses were conducted (Table 3). The results showed no significant difference in the unadjusted (beta = −0.57, 95% CI = −1.14 to 0.00, *p* = 0.057) and adjusted models (adjusted beta = −0.55, 95% CI = −1.15 to 0.05, *p* = 0.080) in terms of the HWES cosmetic appearance score.

### 3.5. Outcome: Patient-Reported Outcomes

Table 2 demonstrates that patients who received tissue adhesives reported higher levels of satisfaction and lower levels of pain compared to those who received conventional sutures, and these differences were statistically significant. To examine patient-reported outcomes between the tissue adhesive and suture groups, we conducted both unadjusted and adjusted logistic regression analyses (Table 3). The results showed that in the unadjusted model, there was a statistically significant difference in increased satisfaction (beta = 1.10, 95% CI = 0.62 to 1.58, *p* < 0.001) and reduced pain (beta = −3.05, 95% CI = −4.17 to −1.93, *p* < 0.001). This significance persisted even after accounting for covariates such as age, sex, BMI, eczema and keloid history, and wound incision length in the adjusted logistic regression model (satisfaction: adjusted beta = 1.13, 95% CI = 0.63 to 1.63, *p* <0.001; pain: adjusted beta = −3.03, 95% CI = −4.15 to −1.90, *p* < 0.001)

### 3.6. Illustrative Clinical Case

Following our statistical analysis, we present a representative clinical case that further illustrates the potential benefits of OCA tissue adhesives.

**Figure 2:**
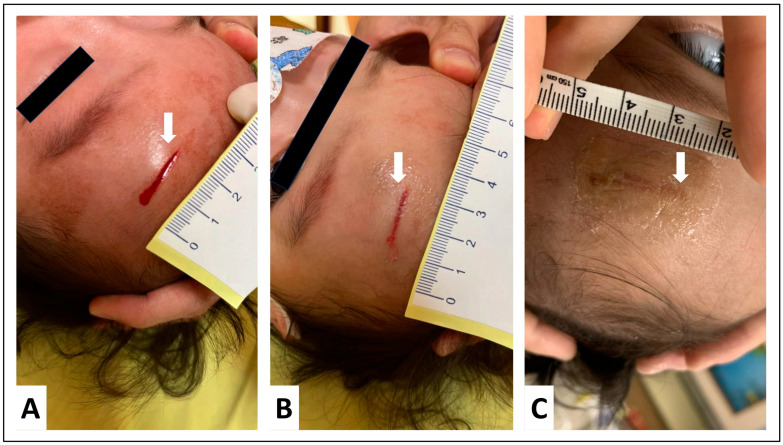
Tissue adhesive treatment in a 4-year-old girl’s facial laceration. (**A**) Initial 2 cm facial laceration (white arrow). (**B**) Wound status at 7-day follow-up, showing significant healing. (**C**) Three months post-treatment, with a Hollander Wound Evaluation Score (HWES) of 0, indicating near-perfect recovery.

A 4-year-old girl with no underlying systemic diseases affecting wound healing presented to the clinic with a 2 cm facial laceration (Figure 2A). OCA tissue adhesives were chosen as the treatment modality over traditional stitches or staples. At the 7-day follow-up, the wound demonstrated remarkable healing, absent of complications such as infection or dehiscence (Figure 2B). Three months post-treatment, a comprehensive assessment using the HWES reflected optimal wound healing, with a score of zero across all evaluation categories, indicating an almost flawless recovery (Figure 2C). The patient’s subjective experience, as captured by a visual analog scale, highlighted an overall satisfaction score of 8 and a pain score of 3, suggesting minimal discomfort during adhesive application and substantial satisfaction with both the procedure and the outcome. This case underscores the efficacy and potential benefits of OCA tissue adhesives, especially in pediatric contexts where patient comfort and cosmetic outcomes are paramount.

## 4. Discussion

In this study, we evaluated the performance of tissue adhesives in comparison to conventional sutures in treating uncomplicated facial lacerations in children. The results demonstrated that tissue adhesives not only yielded similar rates of complications and cosmetic appearance as sutures but also led to enhanced patient satisfaction and diminished pain.

### 4.1. Timing and Methodology of Cosmetic Assessment

The cosmetic appearance was not assessed immediately post-suture removal in our study, given that early appearance is not necessarily indicative of long-term cosmetic results [13]. We focused our assessment three months post-closure, as evidence suggests that a wound’s appearance at this juncture correlates with its cosmetic status after a year [15]. Furthermore, we employed the HWES for this evaluation due to its high concordance among physicians and its alignment with patient assessments [3].

### 4.2. Emerging Preference for Tissue Adhesives: Evidence from Previous Studies

Tissue adhesives have consistently demonstrated fewer wound-related complications and comparable cosmetic outcomes when juxtaposed with traditional sutures. These benefits have been reported in multiple clinical studies and meta-analyses, making tissue adhesives an increasingly popular choice for skin closure in simple traumatic lacerations [16]. Previous research has shown that the use of tissue adhesives for skin closure in cesarean deliveries leads to more favorable cosmetic results compared to skin sutures, without any increase in the incidence of wound disruption or infection [17]. In wound closure after brain surgery, the use of tissue adhesives has been shown to lead to better cosmetic results and increased patient satisfaction compared to conventional sutures, making tissue adhesives a potential and valuable alternative to traditional wound closure methods [18]. The results of a recent retrospective analysis of 492 patients who underwent minimally invasive surgery for colorectal cancer showed that skin adhesive bonds for wound closure resulted in a lower rate of surgical site infection and overall cost for wound care compared to conventional skin stapler techniques [19]. Tissue adhesives offer an expedient and painless application process [20], sidestepping the need for local anesthetic shots, which often induce anxiety in pediatric patients. The elimination of suture removal further simplifies the treatment experience, rendering it less distressing for the child [21]. Its waterproof attributes also confer the advantage of allowing brief water exposure, obviating the need for suture removal.

### 4.3. Potential Risks Associated with Tissue Adhesives

Tissue adhesives are not without their risks, sharing several with traditional sutures, including concerns over scarring, infections, and wound dehiscence [22]. While a systematic review did highlight a marginally increased risk of wound separation with tissue adhesives, our study did not corroborate this finding [23]. Exercising caution is essential, especially around sensitive areas such as the mouth and eyes, where instances of inadvertent closures have been reported [24,25,26]. Notably, our cohorts did not present any allergic reactions or contact dermatitis.

### 4.4. Safety and Toxicity of Tissue Adhesives in Pediatric Facial Lacerations

The application of tissue adhesives, especially in pediatric care, garners significant attention due to children’s delicate skin and the requisite for optimal cosmetic healing. The distinction between the use of butylcyanoacrylate (BCA) and OCA in managing facial laceration wounds among children primarily rests on their chemical constitution and resulting attributes. Contemporary market observations reveal that both BCA and OCA are prevalent ingredients in numerous commercial products. Intriguingly, OCA, with its elongated carbon chain, is associated with reduced heat reactions, irritability, and toxicity compared to its BCA counterpart [27]. This property profile positions OCA as a potentially preferable choice in pediatric care.

Historical literature provides robust evidence affirming the safety of these elongated-chain cyanoacrylate tissue adhesives. Since their introduction in the 1980s, their applications on a multitude of human patients have been recorded without any indication of carcinogenicity. A particular study from this era raised eyebrows, as it reported sarcoma development in rats post-subcutaneous injection with an amplified dose of the n-2-butyl monomer [28]. However, this study’s implications are arguable due to several reasons: the non-standard formulation, extreme dosage, and rats’ known susceptibility to the unique Oppenheimer response when confronted with certain foreign bodies [28]. Moreover, while the by-products of these cyanoacrylates are acknowledged to be histotoxic at elevated concentrations, they are not inherently carcinogenic [29]. Subsequent research and vast clinical applications over the decades have consistently negated any carcinogenic potential in humans, emphasizing the adhesives’ safety profile [30].

### 4.5. Cost-Effectiveness in Wound Management

In the realm of wound closure techniques, understanding the economic implications is crucial. A comprehensive study by Man, S.Y. et al. explored the cost dynamics between the tissue adhesive and conventional suture methods for simple laceration closures in emergency departments [31]. Their cost/consequence analysis unveiled that while tissue adhesives led to greater expenses for the Hospital Authority (216.12 (USD 27.70) as opposed to the conventional suture method at 171.33 (USD 21.96)), they were more cost-effective for patients, incurring lower charges (109.68 (USD 14.06) versus 156.96 (USD 20.12)). This suggests a unique trade-off: tissue adhesives might pose a higher initial cost for healthcare institutions but present an economically favorable option for patients. Therefore, when deliberating on the cost-effectiveness of wound management methods, both institutional costs and patient charges should be considered.

### 4.6. Technical Considerations and Limitations of Tissue Adhesives

It is imperative to underscore that wound closure is but one facet of comprehensive wound management in pediatric patients. There are instances where wounds demand irrigation, debridement, or deeper sutures. Such processes can be labor-intensive, possibly requiring sedation or anesthesia. Tissue adhesives, while quick and relatively painless, may yield suboptimal outcomes if inappropriately deployed [10].

### 4.7. Discussion on Suture Material

In our study, we employed Nylon sutures for wound closure, a choice rooted in the material’s proven track record in medical applications. Nylon, as a non-absorbable synthetic suture material, boasts superior tensile strength and flexibility. Its durability ensures that the suture remains intact during the critical wound-healing phase, especially in regions of the body that experience frequent movement [32].

Moreover, medical-grade Nylon is formulated to be non-toxic, resulting in minimal adverse tissue reactions, thus making it a preferred option in many surgical settings. The biocompatibility of Nylon ensures its safe application, with reduced risks of complications. Over the years, Nylon sutures have become a benchmark in wound closure techniques due to their reliable performance and widespread acceptance in the medical community [33]. Within our study, their use served as the control group, a testament to Nylon’s omnipresence in wound closure contexts.

### 4.8. Limitations

The results of this study should be considered in light of some limitations. Firstly, this was a retrospective cohort study that analyzed data from a database designed to capture a wide range of wound variables. As a result, certain specific details relevant to our study, such as the cause of the lacerations and the time elapsed between injury and treatment, were not recorded. Secondly, the patient population in this study was relatively small compared to a national database that involved multiple institutions. Thirdly, only uncomplicated lacerations with a length of less than 5 cm and a depth of no more than 4–8 mm, which would require 5-0 or smaller nylon sutures for skin closure, were included. Our findings cannot be extrapolated to more complex lacerations or injuries with a more complicated mechanism. Finally, there may have been potential confounding factors that could have affected the results. Further, larger, and randomized controlled studies comparing tissue adhesives with other methods are needed to validate these findings.

## 5. Conclusions

Tissue adhesives offer a convenient and efficient approach to the closure of uncomplicated facial laceration wounds in children. In comparison to sutures, they are associated with reduced discomfort, increased patient satisfaction, and comparable cosmetic outcomes. The incidence of complications, such as dehiscence and infection, is infrequent and mirrors the results achieved with sutures. We found that in the context of managing uncomplicated facial wounds in children, tissue adhesives pose minimal complications and are comparable in efficacy to sutures.

## Figures and Tables

**Table 1:** Characteristics of the study patients.

	All Patients		
	Tissue Adhesive (*n* = 20)	Suture (*n* = 30)	*p* Value
Patient Characteristics			
Age (years)	5.00 ± 3.34	4.77 ± 3.13	0.825
Sex (male), *n*	12 (60.0%)	17 (56.7%)	0.815
Height (cm)	108.85 ± 22.91	107.20 ± 21.82	0.819
Weight (kg)	20.41 ± 13.11	19.44 ± 11.48	0.789
BMI (kg/m^2^)	16.00 ± 2.26	15.90 ± 2.07	0.945
Eczema	3 (15.0%)	3 (10.0%)	0.672
Psoriasis	0 (0%)	0 (0%)	-
Previous soft tissue infection	0 (0%)	0 (0%)	-
Keloid history	1 (5.0%)	3 (10.0%)	0.641
Wound condition			
Site			1.000
Chin	10 (50.0%)	15 (50.0%)	
Eyebrow	2 (10.0%)	3 (10.0%)	
Forehead	7 (35.0%)	10 (33.3%)	
Lower eyelid	1 (5.0%)	2 (6.7%)	
Length of incision	1.48 ± 0.63	1.32 ± 0.47	0.472
Status of tissue adhesive			0.400
Completely sloughed off	1 (5.0%)	0 (0.0%)	
Intact	19 (95.0%)	30 (100.0%)	

Testing by Fisher exact test, Wilcoxon test, or Kruskal–Wallis test, respectively.

**Table 2:** Outcome.

	All Patients		
	Tissue Adhesive (*n* = 20)	Suture (*n* = 30)	*p* Value
Complications			
Wound dehiscence	1 (5.0%)	0 (0.0%)	0.400
Wound infection	1 (5.0%)	2 (6.7%)	1.000
Cosmetic appearance			
HWES score	0.20 ± 0.52	0.77 ± 1.22	0.054
Step-off borders	0 (0.0%)	6 (20.0%)	0.069
Contour irregularities	0 (0.0%)	8 (26.7%)	0.015
Margin separation	3 (15.0%)	1 (3.3%)	0.289
Edge inversion	0 (0.0%)	4 (13.3%)	0.140
Excessive distortion	0 (0.0%)	0 (0.0%)	0.157
Overall appearance	1 (5.0%)	4 (13.3%)	0.636
Patient-reported outcomes			
Satisfaction	8.50 ± 1.00	7.40 ± 0.72	<0.001
Pain	3.25 ± 1.77	6.30 ± 2.10	<0.001

Testing by Fisher exact test, Wilcoxon test, or Kruskal–Wallis test, respectively.

**Table 3:** Univariate and multivariate analysis.

Outcome	Univariate Analysis		Multivariate Analysis	
Complications	Crude-OR (95% CI)	*p*-Value	Adj-OR (95% CI) #	*p*-Value
Wound dehiscence	1.53 (0.09–25.90)	0.770	1.56 (0.08–31.25)	0.773
Wound infection	0.74 (0.06–8.71)	0.809	2.17 (0.08–58.80)	0.645
Cosmetic appearance	Crude-beta (95% CI)	*p*-value	Adj-beta (95% CI) #	*p*-value
HWES score	−0.57 (−1.14–0.00)	0.057	−0.55 (−1.15–0.05)	0.080
Patient-reported outcomes	Crude-beta (95% CI)	*p*-value	Adj-beta (95% CI) #	*p*-value
Satisfaction	1.10 (0.62–1.58)	<0.001	1.13 (0.63–1.63)	<0.001
Pain	−3.05 (−4.17–−1.93)	<0.001	−3.03 (−4.15–−1.90)	<0.001

# All results of Adj-OR were adjusted by age, sex, BMI, eczema, keloid history, and length of incision.

## Data Availability

The data supporting the findings of this study are available upon request. Interested parties can contact the corresponding author for access.
